# Unveiling the Mechanism for the Split Hysteresis Loop in Epitaxial Co_2_Fe_1-x_Mn_x_Al Full-Heusler Alloy Films

**DOI:** 10.1038/srep18615

**Published:** 2016-01-06

**Authors:** X. D. Tao, H. L. Wang, B. F. Miao, L. Sun, B. You, D. Wu, W. Zhang, H. P. Oepen, J. H. Zhao, H. F. Ding

**Affiliations:** 1 National Laboratory of Solid State Microstructures and Department of Physics, Nanjing University, 22 Hankou Road, Nanjing 210093, P. R. China; 2 State Key Laboratory of Superlattices and Microstructures, Institute of Semiconductors, Chinese Academy of Sciences, P.O. Box 912, Beijing 100083, China; 3 Institut für Angewandte Physik, Universität Hamburg, Jungiusstraße 11, Hamburg 20355, Germany; 4 Collaborative Innovation Center of Advanced Microstructures, Nanjing University, 22 Hankou Road, Nanjing 210093, P. R. China

## Abstract

Utilizing epitaxial Co_2_Fe_1-x_Mn_x_Al full-Heusler alloy films on GaAs (001), we address the controversy over the analysis for the split hysteresis loop which is commonly found in systems consisting of both uniaxial and fourfold anisotropies. Quantitative comparisons are carried out on the values of the twofold and fourfold anisotropy fields obtained with ferromagnetic resonance and vibrating sample magnetometer measurements. The most suitable model for describing the split hysteresis loop is identified. In combination with the component resolved magnetization measurements, these results provide compelling evidences that the switching is caused by the domain wall nucleation and movements with the switching fields centered at the point where the energy landscape shows equal minima for magnetization orienting near the easy axis and the field supported hard axis.

Magnetic anisotropy is one of the fundamental properties of magnetic materials which governs their applications. According to the symmetry of ferromagnet, magnetic anisotropies can be classified into twofold anisotropy (also called uniaxial anisotropy) *K*_2_, fourfold anisotropy *K*_4_, and higher order anisotropies. Typically, they are mixed in one material. The coexistence of *K*_2_ and *K*_4_ is the most common case for magnetic systems mainly due to the competition between the crystalline anisotropy and the uniaxial anisotropy induced by lattice mismatch, miscut and inclined deposition etc. Several interesting phenomena such as the split hysteresis loop (Fig. 1), and the field-induced spin reorientation transition (SRT) have been observed^1^,^2^,^3^,^4^,^5^,^6^,^7^,^8^,^9^,^10^,^11^,^12^,^13^,^14^. In order to obtain the magnetic anisotropy constants quantitatively from the hysteresis loop, several models have been proposed^1^,^4^,^6^. Weber *et al.*^1^, pointed out the uniaxial and fourfold anisotropies can be directly given by the split field *H*_*s*_ and the linear slope *k* of the split hysteresis loop at zero field [For the definition of *H*_*s*_ and *k*, see Fig. 1]. Through minimizing the in-plane free energy with respect to the angle between magnetization and magnetic field and assuming 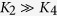, the authors derived that 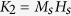 and 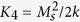, where *M*_*s*_ is the saturation magnetization. Dumm *et al.*^4^, recognized that *K*_2_ and *K*_4_ depend on both *k* and *H*_*s*_. Meanwhile, Oepen *et al.*^6^, analyzed the split loops in the framework of the field-driven SRT. Their results showed that the switching fields in the split loops are directly related to the SRT and thus *K*_2_ and *K*_4_ are obtained by the switching fields and *k*. The different models have been adopted by many groups^11^,^15^,^16^,^17^,^18^,^19^,^20^,^21^,^22^,^23^,^24^. However, they have not been cross-checked with additional magnetic anisotropy sensitive techniques such as ferromagnetic resonance (FMR) and magnetic torque measurements.

In this paper, we identified the model which best describes the system by comparing the uniaxial anisotropy field 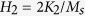 and fourfold anisotropy field 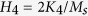 obtained with FMR and vibrating sample magnetometer (VSM) measurements on the Co_2_Fe_1-*x*_Mn_*x*_Al films with different Mn concentrations. Co_2_Fe_1-*x*_Mn_*x*_Al is a kind of Co-based full-Heusler alloys, which possess high spin polarization and have great potential in spintronics application^25^,^26^,^27^,^28^,^29^,^30^,^31^,^32^,^33^,^34^. Interestingly, electronic structure calculations have revealed that Co_2_MnAl can retain half-metallic property for different levels of Fe doping^35^,^36^. The magnetic anisotropy however shows strong variations on concentration which mainly due to a competition between uniaxial and four-fold anisotropies^37^,^38^. Thus, it provides an interesting system to crosscheck the different models used in the split hysteresis analysis besides the fundamental interest in the exploration of the concentration dependent properties. We first measured the hysteresis loops with VSM and calculated the anisotropy fields from the split loops using different models. The results are quantitatively compared with the measured values utilizing FMR which allows us to identify the most suitable model used in the split loop analysis. The underlying physics is discussed. And the concentration dependent important material parameters such as effective magnetization, anisotropy fields, and damping constants are also given.

## Results

### Controversy over the analysis for the split hysteresis loop

**Figure 1:**
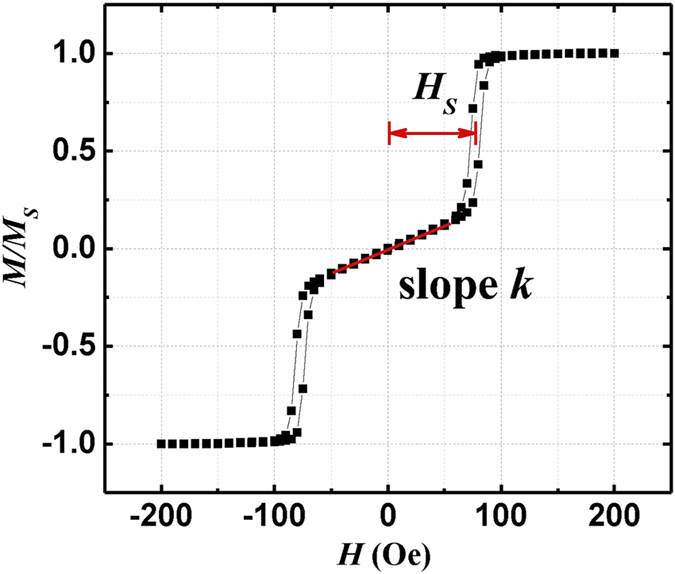
Typical split-hysteresis loop obtained using VSM on a 45 nm epitaxial Co_2_FeAl film along the [11-0] direction at room temperature. *H*_*s*_ and *k* are the split field and the slope at the zero field, respectively.

Figure 1 shows a typical split hysteresis loop of a 45 nm Co_2_FeAl film measured by VSM with the magnetic field being applied within the sample plane and along the [110] direction. A discontinuity appears around *H*_*s*_. This discontinuity is assumed to be the consequence of the superposition of the uniaxial and the fourfold anisotropy in the case that the hard axis of the uniaxial anisotropy coinciding with an easy axis of the fourfold component^1^,^4^,^6^. With the measured split loops, we calculated *H*_2_ and *H*_4_ utilizing the three models mentioned above. The results are listed in Table 1 for Co_2_FeAl and Co_2_Fe_0.7_Mn_0.3_Al films. Both films have the same thickness of 45 nm. We can find that the results obtained with different models are significantly different. This raises an interesting question which model describes the magnetic anisotropies of the system best.

### FMR measurements and analysis

**Figure 2:**
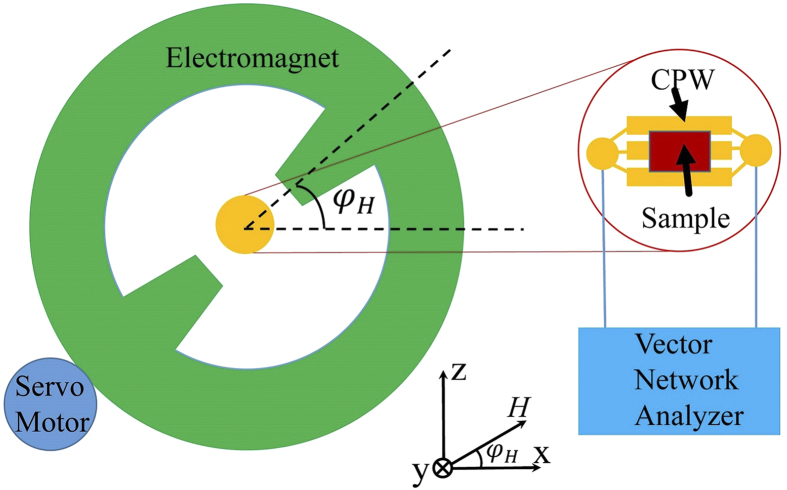
Sketch of the FMR measurement configurations in our experiment. The sample was sticked on a CPW fixture to be excited by a *rf* magnetic field. VNA is used to generate microwave magnetic field and detect the *S*_21_ parameters. The external magnetic field can be rotated in-plane by a servo motor.

**Figure 3:**
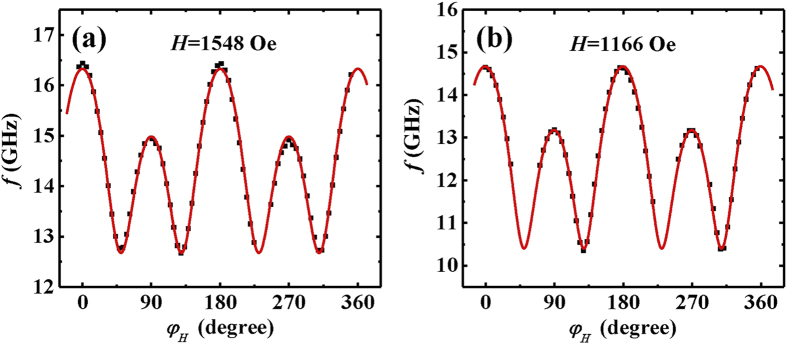
Angular-dependent ferromagnetic resonance frequency with a constant magnetic field applied parallel to the film for the sample. The magnetic field is 1548 Oe for (**a**), while 1166 Oe for (**b**). The solid line is fitting results with the parameters in Table 2.

To obtain independent results for crosschecking the different models, we performed angular dependent measurements utilizing FMR. The samples were positioned on a coplanar waveguide (CPW) fixture with the [110] direction of the film parallel to the *x*-direction as sketched in Fig. 2. The CPW is connected with a Vector Network Analyzer (VNA), which generates microwave with tunable frequencies. The VNA also detects the *rf* signal via the change of the forward transmission coefficient in scattering parameters, *S*_21_. The external magnetic field *H* is applied within the film plane. The orientation of magnetic field is controlled by a servo motor with high accuracy of positioning (error margin <0.15°). We note that all the graphs are plotted with the raw data without any mathematical smoothing. The magnitude of the magnetic field is first set to be 1548 Oe, which is larger than the saturation field. The microwave frequency is swept to find the resonance condition where the maximum absorption occurs. Rotating the magnet yields an angular-dependent resonance frequency for Co_2_FeAl which is plotted in Fig. 3(a). From the plot of the angular dependence one can easily identify twofold and fourfold symmetries with the maximum frequency located at 0° and 180°. Since the resonance frequency is proportional to the effective magnetic field, the maximum (minimum) value is found when the field is parallel to the easy (hard) axis. The angular dependence of the resonance frequency immediately shows that the easy axis is along the [110] direction, consistent with the hysteresis loop. The uniaxial easy axis coincides with one of the fourfold axes and is along the [110] direction. Therefore, [11-0] bears the easy character of the fourfold cubic anisotropy and the hard character of the uniaxial anisotropy, which causes the split hysteresis loop (Fig. 1).

By summing up the Zeeman, the demagnetization and the anisotropy energy densities, the total free energy density of the system can be written as:




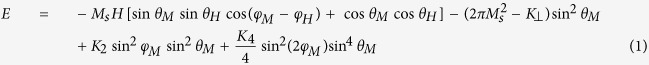




where 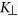, *K*_2_ and *K*_4_ are the out-of-plane uniaxial, in-plane uniaxial and fourfold anisotropy constants, respectively. And 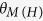 are the angles between *M*(*H*) and the axis perpendicular to the film plane (*y-*axis), while 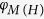 are the in-plane angles between *M* (*H*) and the easy axis (*x*-axis), see Fig. 2. Here the exchange energy and the higher-order anisotropy energies are neglected since the magnetic field used in the measurements is larger than the saturation field and the measured data do not show higher-order anisotropy. According to the FMR theory^39^, the resonance frequency *f* of the precession can be obtained as 
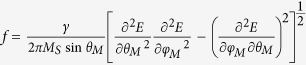
. In our measurements, the magnetic field is applied within the film plane, *i.e.*, 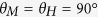. Therefore, it can be simplified as:




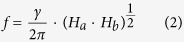




where 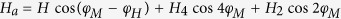 and 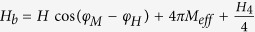 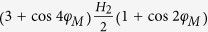. *γ* is the gyromagnetic ratio. 
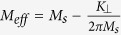
 is the effective magnetization of the sample. According to equation (2), we can fit the angular dependent measurements. The fitted result is plotted as the red line in Fig. 3(a). The plot reproduces the data very well. The best fitting parameters are listed in Table 2. The fitting yields 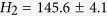 Oe and 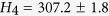 Oe for Co_2_FeAl. The obtained effective magnetization and g are 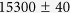 Oe and 2.0, respectively. On account of the complexity of multi-parameters fitting, we repeat the same measurements at a field with different amplitude, *i.e.*, 1166 Oe to double-check the fitted results. The experimental data and the fitted curve are shown in Fig. 3(b). The fitting yields the same results within the error margin of experiments (see Table 2). The quantitative agreement evidences that the method used in the angular dependent FMR measurements and analysis is accurate and with high reproducibility.

### Comparison of FMR and VSM measurements along different directions

**Figure 4:**
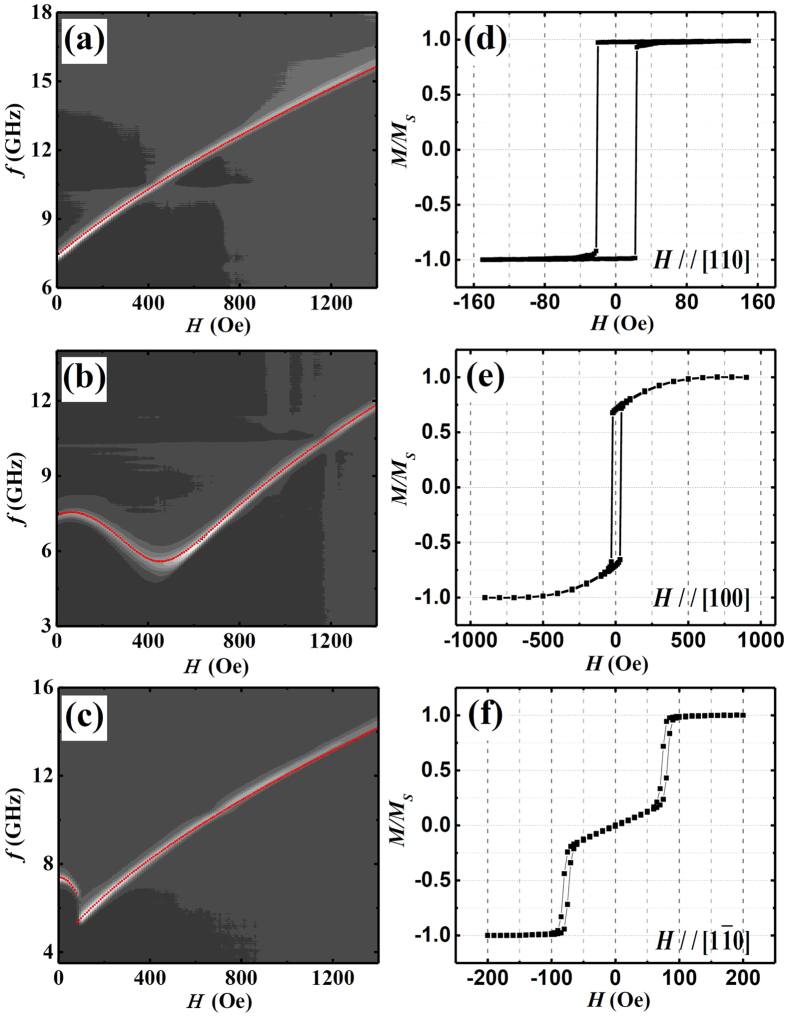
Left column (a–c) are 2D gray scale mapping of the *S*_21_ absorption signal as function of the frequency and H with the magnetic field along the [110], [100],[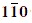] directions, respectively. The white indicate the position of FMR. The red lines are fitting results with the parameters in Table 2. Right column (**d–f**) are the corresponding longitudinal hysteresis loops of Co_2_FeAl film measured by VSM at RT.

To further check the accuracy of the fitted results, we also performed field dependent FMR measurements along different directions. In the left column of Fig. 4, we show the two dimensional (2D) gray scale mapping of the *S*_21_ signal of the microwave absorption measured via VNA as a function of the microwave frequency *f* and magnetic field *H* with the field applied along the [110], [100] and [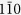], respectively. The right column displays the corresponding hysteresis loops obtained via VSM. In the FMR measurements we swept the magnetic field from ~ +1400 Oe to zero. The brightness of the grayscale shows the amplitude of the absorption and the brightest spots represent the position of FMR. Since the anisotropy fields cause additional contributions to the torque on the magnetization, FMR spectrum exhibits different behavior in these three directions. The easy axis of magnetization is along the [110] direction, as can be recognized from the rectangular shape of the hysteresis loop in Fig. 4(d). The data for the field along the [110] direction [Fig. 4(a)] show a typical FMR spectrum. It can be easily fitted with Kittel formula (red curve). In this case, the magnetization precesses around the direction of external magnetic field. The curve along the [100] direction, Fig. 4(b), is however more complicated. With the increase of the magnetic field, the resonance frequency first decreases then increases after an inflection field at ~450 Oe. Consequently, two resonance modes at different fields can be obtained for a given frequency. For instance, a 7 GHz microwave field causes resonance at either 200 Oe or 700 Oe. From Fig. 4(e), it is obvious that [100] is the hard axis of the sample and it saturates at the field higher than 500 Oe. The remanence is about 0.7 of the saturation value which can be easily understood as the field is applied 45° with respect to the easy axis, i.e., the [110] direction and the magnetization is expected to align close to the easy axis at low external field. With the increasing of the magnetic field, the magnetization will align along the field direction eventually. In such case, *φ*_*M*_ is no longer constant, but is determined by the total energy minimum of equation (1). In fact, it changes from 45° to 0°. From equation (2), we see that the resonance frequency is proportional to 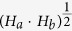 with *H*_*b*_ consisting of four terms, 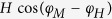, 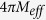, 
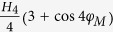
 and 
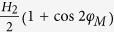
. Among them, 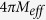 is a constant of ~15300 Oe and is much larger than the applied field and the anisotropy fields. Therefore, *H*_*b*_ shows only a weak dependence on field. *H*_*a*_ consists of three terms, 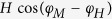, 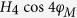 and 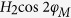. When the field is not applied along the easy axis, the terms 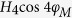 and 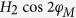 decrease with increase of the magnetic field as the magnetization deviates from the easy axis, while 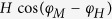 behaves oppositely. Hence the resonance frequency is determined by the competition between anisotropy fields and magnetic field. As listed in Table 2, both *H*_2_ and *H*_4_ are comparable with *H* at low magnetic field which can lead to a decrease of resonance frequency *f* with increasing field in a certain field range, as shown in Fig. 4(b). Taking the same parameters in the angular dependent FMR measurements as in Fig. 3 and using equations (1) and (2), we have computed the field dependent *f* (red curve) and found that it agrees well with the experimental data. Similarly, we can understand the field dependence of *f* for the field applied along the 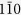] direction [Fig. 4(c)] which is also not an easy axis. The resonance frequency decreases with increasing the field up to 80 Oe. After a sharp drop, the spectrum increases with field as expected. The experimental data can be well produced with the calculation (red curve) except the jump at ~80 Oe. We note that the hysteresis loop along the [ 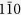 ] direction shows the similar behavior. At large field, the magnetization *M* is forced by the field *H* to align along the [ 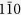 ] direction. At zero field *M* is oriented along the easy axis and perpendicular to *H*. Depending on either increase or decrease of the field, the magnetization curve shows two discontinuities at two different switching fields around *H*_*s*_. We further performed the field dependent FMR measurements with ascending field and found that the jumping field in the FMR occurs at exactly the same field as the switching field in the field ascending branch of hysteresis loop. The one-to-one correspondence identifies that the origin of the jump in the field dependent FMR measurements shown in Fig. 4(c) is the magnetization switching. We emphasize that all the three resonance curves shown in Fig. 3(a–c) can be well reproduced with the parameters in Table 1 according to equations (1) and (2) [red lines in Fig. 3(a–c)] once again, proving the validity of the method used for anisotropy field analysis in the FMR measurements.

### Underlying mechanisms for the split hysteresis loop analysis

Comparing the results obtained via FMR with the values derived from the VSM measurements utilizing different models (Table 1), we recognize that the values obtained with the model by Dumm *et al.*^4^, gives the closest agreement. It is not too surprising to find that the model by Weber *et al.*^2^,^3^, could not yield similar results since their model is applicable only for 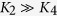. In the model proposed by Oepen *et al.*^6^, the field driven reorientation for a strong uniaxial behavior is discussed. The assumption of the model is that without field the only minimum of the energy landscape appears for magnetization parallel to one axis only while the field applied along the hard axis drives the system through a state of coexisting phases (or metastability^40^,^41^,^42^). The latter scenario can be found only for 
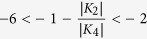
. We note here that, in the original paper, the free energy is expressed as 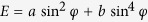 and a translation of 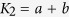 and *K*_4_ = *b* is therefore required for comparison. The main assumption of the model is that in descending field the magnetization switches back into the easy axis when the local minimum created by the field is erased. With values of the anisotropy obtained via FMR it is evident that 
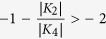
 and thus the prerequisite not fulfilled.

**Figure 5:**
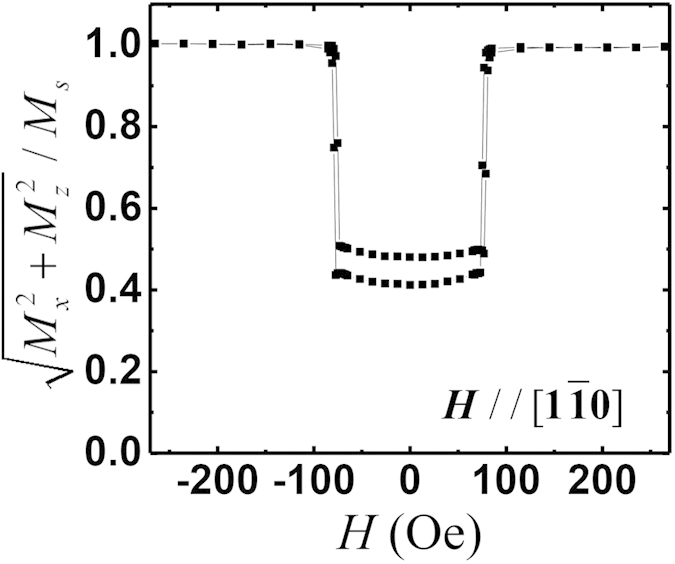
The normalized value of the magnetization calculated from the two in-plane components. The magnetic field is applied along the [ 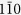 ] direction. Due to the strong shape anisotropy, the perpendicular component is negligible.

In the system studied here, however, the starting point (zero field) is situated within the range of metastability (see Fig. 4b in ref. 41) as 
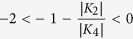
. In lowest order approximation the field applied along the harder axis weakens the *K*_2_ contribution. Trespassing the range of metastability means to change the depth of the two coexisting minima. Dumm *et al.*^4^, considered the switching to appear around the point where the magnetic field causes an energy degeneracy of the two states. Apparently the latter is the appropriate assumption to describe the situation in the system investigated here. The key issue to the understanding is the fact that the anisotropies as well as the potential well are very small. As the saturation magnetization is high even the minor values of the in-plane shape anisotropy can have an impact. At the edges the demagnetizing fields can cause a local reorientation of the magnetization which causes an instantaneous reversal of magnetization via domain wall movement on further decrease of field strength. This is confirmed by the simultaneously obtained magnetization along different directions (not shown). The normalized value of the magnetization calculated from the individual components along different direction shows a strong decrease around the switching fields (Fig. 5) evidencing that the switching is caused by the domain wall nucleation and domain wall movements^43^.

In ref. 4 as well as in ref. 6 a reversible rotation near the zero field in the potential that is determined by the sum of two- and fourfold anisotropy contribution is assumed. In case of strong shape anisotropy the magnetization reversal appears fully within the sample plane, the total energy of the system [equation (1)] can be simplified by taking 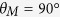. As the magnetic field is applied along the [ 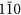 ] direction, i.e., 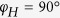, an analytic expression for *H*(*m*) can be derived by minimizing the total energy *E, i.e.,*




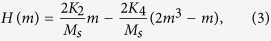




where 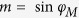 is the normalized magnetization component along the applied field direction. By differentiating equation (3), one can obtain the inverse slope of *m*(*H*) for *m* = 0 as




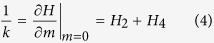




The linearization is a valid approximation which is proven experimentally by the split hysteresis loop.

**Figure 6:**
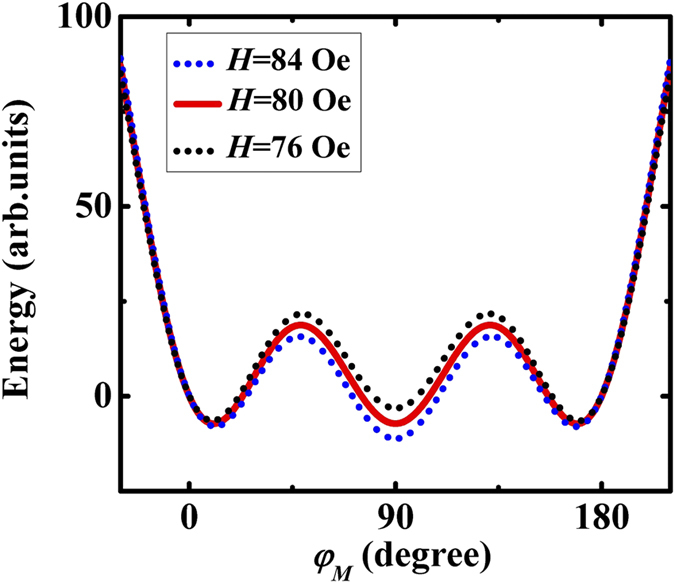
Plot of the angle dependent free energy with a magnetic field along the [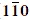] direction. *H*_2_ equals to 145.6 Oe and *H*_4_ equals to 307.2 Oe. The magnetic field is along 90° and the easy axis is 0° and 180°. The magnetic field is set to be 84, 80, and 76 Oe, respectively.

Dumm *et al.* further assumed that the energies are the same for two magnetization configurations at split field *H*_*s*_ in the split loop, 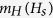 and 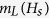. Plotting the angle dependent free energy with a magnetic field along the [ 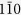 ] direction for 80 Oe, i.e. the field value of the jump in the FMR measurement, and 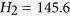 Oe and 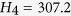 Oe (FMR results) we obtain three local energy minima around 0°, 90° and 180° values (see Fig. 6). The minima have the same value at 80 Oe while for fields that are either smaller (76 Oe) or larger (84 Oe), the local minimum at 90° is higher or lower than the other two minima. Hence the FMR data verify the assumption of equal energy states at the switching field. We note that *H*_*s*_ = 77 Oe, the small variance with 80 Oe may origin from the error bar of the measurements. Dumm *et al.*^4^, made reasonable settings for the magnetization component along the hard axis of 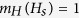 and 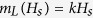. The latter in an extrapolation utilizing the zero-field slope which is a good assumption as long as 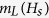 is small. For the sake of a more general treatment, we propose to take the measured value of 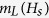 and from the hysteresis curve. Combining it with equation (4), the twofold and fourfold anisotropy fields can be derived as:




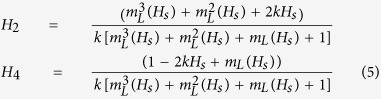




With equation (5), we can obtain the twofold and fourfold anisotropy fields from *H*_*s*_, 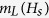 and slope *k*. The obtained results, 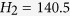 Oe and 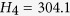 Oe, are consistent with the fitted data from the FMR measurements.

### Confirmation with different Mn concentration

**Figure 7:**
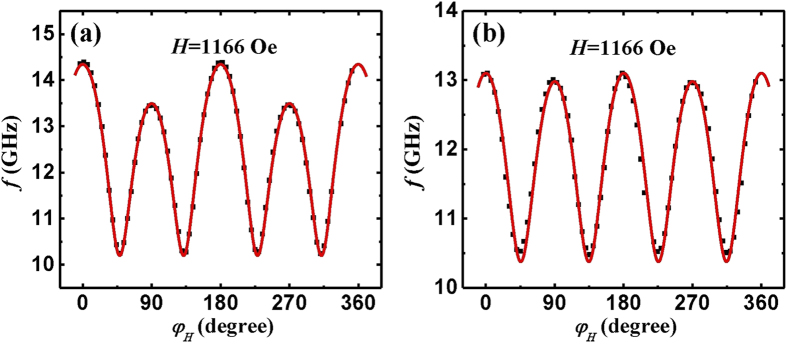
Angular-dependent ferromagnetic resonance frequency with a constant magnetic field applied in the plane of films from (a) *x* = 0.3 and (b) *x* = 0.7. H = 1166 Oe.

Above, we have investigated the magnetic properties of Co_2_FeAl film via FMR and VSM measurements. In the following, we continue to discuss the property variation with different Mn doping, *i.e.*, Co_2_Fe_0.7_Mn_0.3_Al and Co_2_Fe_0.3_Mn_0.7_Al. Figure 7(a,b) present the angular dependent FMR measurements. They can be fitted very well with equation (2) and the fitted twofold and fourfold anisotropy fields are listed in Table 2. For Co_2_Fe_0.7_Mn_0.3_Al, the fitted results agree very well with VSM measurements using the analysis mentioned above. The good agreement once again proves the validity of the model used in the split loop analysis. From the angular dependent FMR measurements in Fig. 7(b), we can find that Co_2_Fe_0.3_Mn_0.7_Al shows almost pure fourfold symmetry. Meanwhile, hysteresis loops along the [110] and 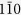 directions become similar. Due to the weak twofold anisotropy, the VSM measurements for Co_2_Fe_0.3_Mn_0.7_Al did not show any split hysteresis loop. Therefore, the quantitative comparison between FMR and VSM measurements is not applicable. Besides, we found that the effective magnetization decreases from 15300 Oe to 14273 Oe and twofold anisotropy field decreases from 145 Oe to 12 Oe with the Mn composition increasing from 0 to 0.7. Recent X-ray magnetic circular dichroism measurements show that Co, Fe, Mn all exhibit net ferromagnetic states and contribute ferromagnetism to the films^37^. Since the magnetic moment of Mn atom is generally larger than that of Fe atom, it would be expected that the magnetization of the system would increase with increasing Mn concentration if Mn, Fe and Co atoms are completely ferromagnetic coupled. Our FMR measurements, however, show the opposite behavior. This strongly suggests that there must be antiferromagnetic interaction among the system. The evolution of *H*_2_ and *H*_4_ with the changing Mn concentration could be associated with the competition between ferromagnetic Ruderman–Kittel–Kasuya–Yoshida exchange and antiferromagnetic superexchange, as reported by Şaşıoğlu *et al.*^44^,^45^, Through the FMR linewidth measurements, we also obtain the damping factor of samples with different Mn concentration. For Co_2_FeAl, the damping factor is 7.7 × 10^−3^, while for Co_2_Fe_0.7_Mn_0.3_Al and Co_2_Fe_0.3_Mn_0.7_Al, this value decreases to 6.5 × 10^−3^ and 5.9 × 10^−3^, respectively.

## Summary

Combining VSM and FMR measurements on the full-Heusler alloy Co_2_Fe_1-*x*_Mn_*x*_Al epitaxially grown on GaAs(001), three different models for the interpretation of split hysteresis loop are checked. The most suitable model is identified as the one that assumes the switching fields centered at the point where the energy landscape shows equal minima near the easy axis and the field supported hard axis. Our studies reveal that *H*_2_ decreases rapidly with increasing Mn concentration and almost vanishes at *x* = 0.7, while *H*_4_ shows much less concentration dependence. The decreasing effective magnetization with adding Mn component strongly suggests the existence of antiferromagnetic coupling among the system.

## Methods

The Co_2_Fe_1-*x*_Mn_*x*_Al samples were prepared by molecular-beam epitaxy on GaAs (001) at 553 K. All the films have the same thickness of 45 nm and the Mn composition *x* varies from 0 to 0.7. Before being taken out of the ultrahigh vacuum chamber, the films were protected by 2 nm of aluminum capping layer. The crystal structure and the quality of order were analyzed by double-crystal X-ray diffraction as described previously^27^. The hysteresis loops along different directions were obtained via VSM. The FMR measurements were performed with a Vector Network Analyzer, which generates microwave with tunable frequencies (20 MHz to 20 GHz). The VNA also detects the *rf* signal via the change of the forward transmission coefficient in scattering parameters, *S*_21_. The external magnetic field *H* was applied within the film plane. The orientation of magnetic field was controlled by a servo motor with high accuracy of positioning (error margin <0.15°).

## Additional Information

**How to cite this article**: Tao, X. D. *et al.* Unveiling the Mechanism for the Split Hysteresis Loop in Epitaxial Co_2_Fe_1-x_Mn_x_Al Full-Heusler Alloy Films. *Sci. Rep.* **6**, 18615; doi: 10.1038/srep18615 (2016).

## Figures and Tables

**Table 1:** The obtained twofold and fourfold anisotropy fields from split hysteresis loop with different models proposed by different groups.

		Weber *et al.*^1^	Dumm *et al.*^4^	Oepen *et al.*^6^
*Co*_*2*_ *FeAl*	*H*_2_(Oe)	154	140.4	259.7
	*H*_4_(Oe)	444.4	304.1	184.7
*Co*_*2*_ *Fe*_*0.7*_ *Mn*_*0.3*_ *Al*	*H*_2_(Oe)	92.2	87.3	239.5
	*H*_4_(Oe)	434.8	347.5	195.4

**Table 2:** The best fitting results of the magnetic parameters for the FMR measurements and anisotropy fields obtained from hard-axis loops.

*x*	0		0.3	0.7	Method
*H* (Oe)	1548	1166	1166	1166	FMR
*g*	2.0 (0.01)	2.0 (0.01)	2.0 (0.01)	2.0 (0.01)	
*M*_*eff*_(Oe)	15300 (40)	15320 (30)	14800 (40)	14273 (40)	
*H*_2_(Oe)	145.6 (1.8)	146 (1.5)	86.7 (2)	12 (0.5)	
*H*_4_(Oe)	307.2 (4.1)	306.1 (5)	340.7 (3.5)	257.1 (2.8)	
*H*_2_(Oe)	140.5 (2)		85.3 (4)	N.A.	VSM
*H*_4_(Oe)	304.1 (3)		349.5 (6)		

The numbers in the parentheses are the experimental error margins.
